# A 92‐year‐old patient with thoracic empyema successfully treated by CT‐guided insertion of a pigtail catheter

**DOI:** 10.1002/rcr2.1162

**Published:** 2023-05-16

**Authors:** Kohei Fujita, Masataka Hirai, Zentaro Saito, Takanori Ito, Takuma Imakita, Issei Oi, Osamu Kanai, Hiromasa Tachibana, Tadashi Mio

**Affiliations:** ^1^ Division of Respiratory Medicine, Centre for Respiratory Diseases National Hospital Organization Kyoto Medical Centre Kyoto Japan

**Keywords:** drainage, elderly patient, empyema, pigtail catheter, pyrothorax

## Abstract

We present a case report of a 92‐year‐old patient with thoracic empyema, who was successfully treated via CT‐guided insertion of a pigtail catheter. The advanced age of the patient often poses challenges in managing pyothorax due to limited physical activity and cognitive decline stemming from decreased activities of daily living. In instances where thoracic drainage is not feasible, the course of treatment is protracted and the prognosis is poor. Our case report exemplifies the successful treatment of pyothorax in a geriatric patient via CT‐guided insertion of a pigtail catheter. We believe that this educational case serves as a testament to the fact that even the most aged patients can be successfully treated with resourcefulness.

## Graphical abstract

We present a case report of a 92‐year‐old patient with thoracic empyema, who was successfully treated via CT‐guided insertion of a pigtail catheter. We believe that this educational case serves as a testament to the fact that even the most aged patients can be successfully treated with resourcefulness.

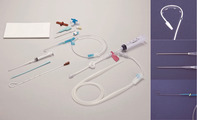

## INTRODUCTION

Thoracic empyema frequently manifests in patients with compromised host immunity, including those with type 2 diabetes mellitus, malignancies, and autoimmune disorders.^1^, ^2^ Antimicrobial therapy alone is often insufficient to manage thoracic empyema, one of the most persistent respiratory infections. Since the 1990s, a gradual rise in pyothorax has been documented in the UK and USA, as well as in Japan, where factors such as an aging population and increased immunosuppressive therapy may be responsible for this trend.^1^, ^2^, ^3^ As a means of source control, thoracic drainage therapy is frequently necessary. However, due to cognitive decline and diminished activities of daily living (ADL) in elderly individuals over 80 years of age, this drainage therapy can be highly invasive and problematic. In light of this, we present a case report demonstrating the successful treatment of thoracic empyema in a 92‐year‐old patient through the use of a minimally invasive pigtail catheter drainage technique, which we believe offers significant educational value.

## CASE REPORT

**FIGURE 1:**
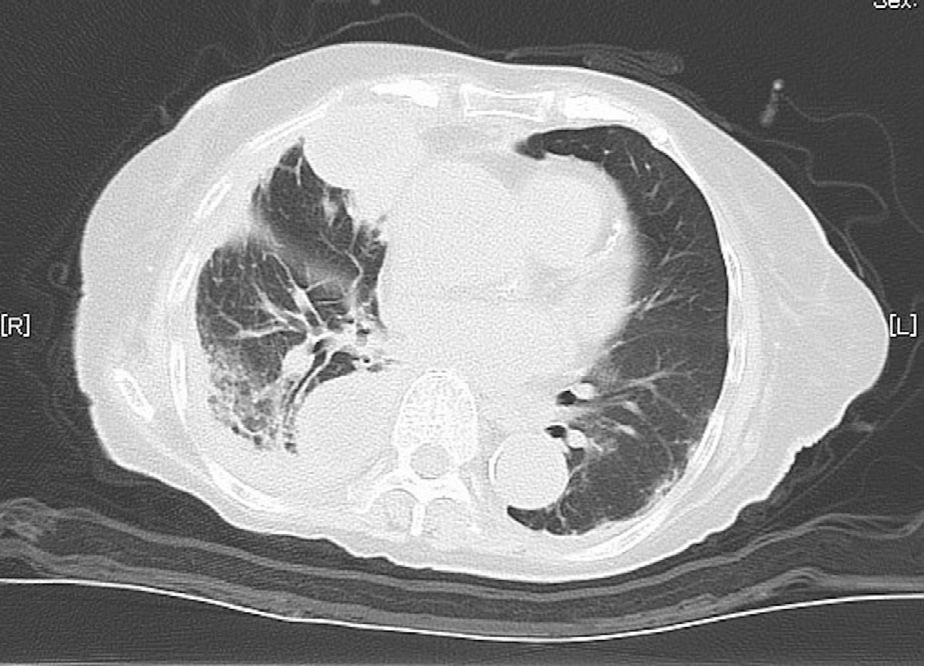
Chest CT scan images upon admission reveal the presence of a pyothorax cavity located in the lower lobe of the right lung, extending from near the spine to the base of the right lower lobe.

A 92‐year‐old female patient was admitted to our hospital due to persistent fever lasting 2 weeks. The patient had been residing in a geriatric care facility for an extended period. Chest CT imaging revealed the presence of multiple loculated empyema in the right lung (Figure 1). The patient required assistance with daily living activities, including eating, and was suffering from dementia and short‐term memory problems. Due to physical conditions and anatomical considerations, thoracoscopy for pyothorax rupture and thoracic drainage with a large drain were deemed unacceptable on admission as the pyothorax cavity was difficult to puncture under bedside thoracic ultrasonography. As a result, an initial decision was made to monitor the patient's condition and administer broad‐spectrum antimicrobials.

**FIGURE 2:**
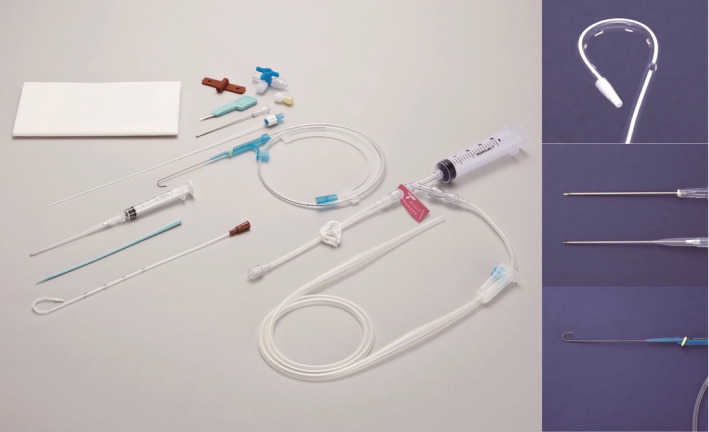
Pigtail catheter kit used in this case. Argyle™ Fukuroi Aspiration Seldinger kit (Pigtail 8Fr 2.7 mm × 20 cm, Cardinal Health, Inc.). All images licensed for use in this case.

**FIGURE 3:**
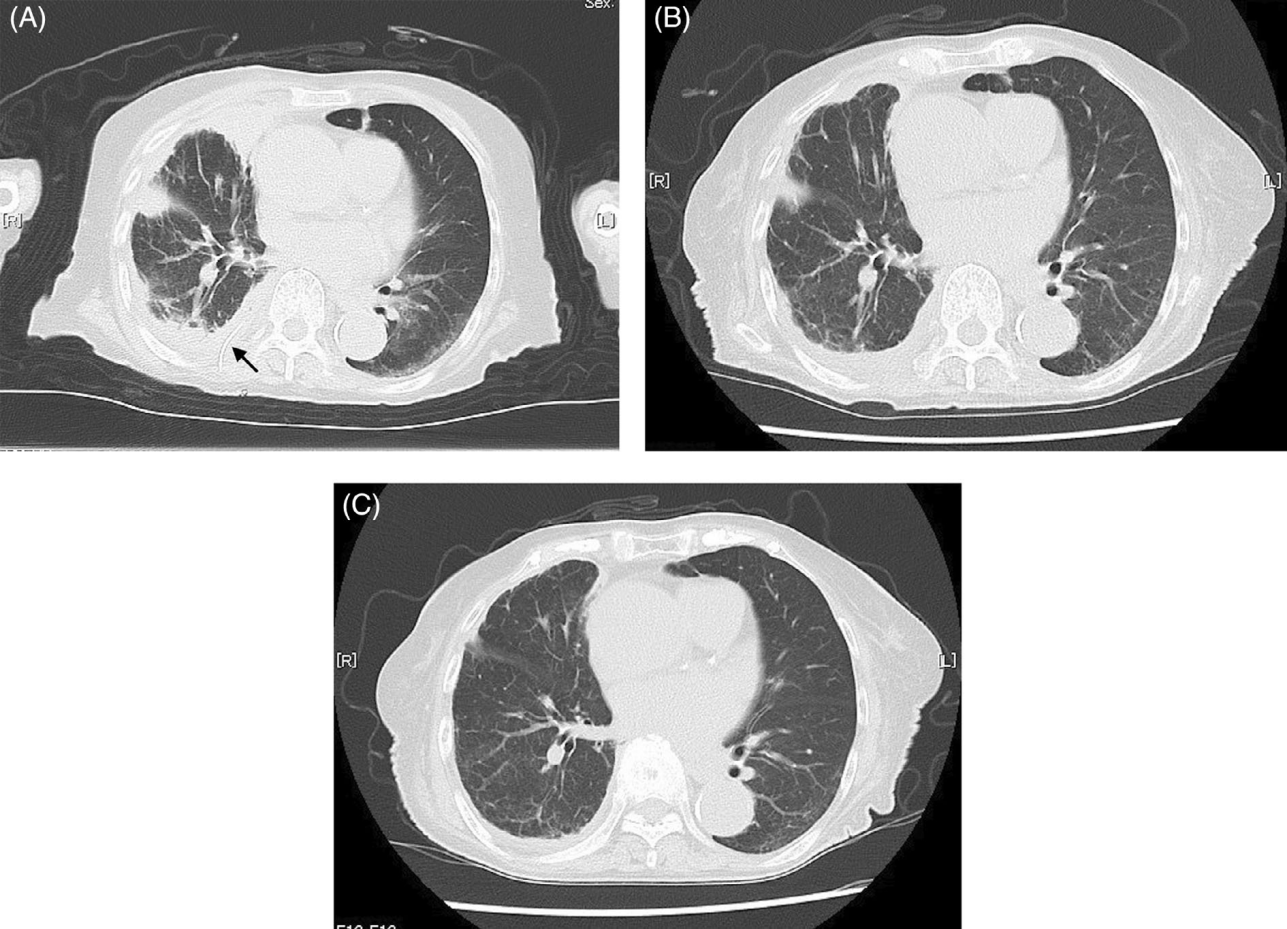
Chest CT scan images captured during pigtail catheter insertion and after drainage. The pigtail catheter was inserted into the cavity of the right lower lobe. (A: pigtail catheter is indicated by black arrow) Subsequent CT images taken 1 month (B) and 2 months (C) after treatment reveal a marked reduction in the pyothorax cavity, leaving only a trace.

After 10 days of antimicrobial treatment, there was no reduction in the lung pyothorax cavity, and the patient continued to experience persistent fever. Consequently, it was decided that pus removal was necessary, and drainage was performed. However, given the patient's advanced age, dementia, and difficulty in following healthcare professional instructions, inserting a large drainage tube was deemed challenging. As a result, an 8 Fr pigtail catheter was inserted under CT guidance due to the anatomical location of the pyothoracic cavity being close to the spine and difficult to access under echo‐guidance. We used Argyle™ Fukuroi Aspiration Seldinger kit (Pigtail 8Fr 2.7 mm × 20 cm, Cardinal Health, Inc.) as shown in Figure 2. The first step is to delineate the lesion with CT and measure the distance to the lesion. Next, with the real‐time CT image projected, the puncture needle is advanced to the pus, the inner tube is removed when it reaches the inside of the pus, and the catheter is implanted. The catheter is fixed after confirming that the pus returns manually. Greyish‐white pus was drained, and the pyothoracic cavity was found to have decreased in size (Figure 3A). The pigtail catheter was left in place for 1 week, during which time negative pressure suction drainage was performed daily, with the route being flushed with heparin. Antimicrobial treatment was administered for a total of 2 weeks before being terminated.

At the end of the third week, the patient was discharged and returned to the original geriatric care facility. A follow‐up CT scan was performed 1 month and 2 months after the insertion of the pigtail catheter, revealing that the pyothorax cavity had nearly disappeared, and the right pyothorax was mildly relieved (Figure 3B, C).

## DISCUSSION

In compliance with established guidelines, early pyothorax is initially treated with antimicrobial therapy and thoracic drainage.^1^, ^2^ If the response to initial treatment is inadequate, further invasive procedures such as thoracoscopy may be warranted. However, in practical settings, test puncture and drainage can be challenging due to the anatomical positioning of the pyothorax cavity. This difficulty is compounded in elderly patients who are bedridden, have communication difficulties, or exhibit poor ADL, rendering invasive procedures even more challenging. Such patients may continue with antimicrobial administration alone, resulting in prolonged hospitalization and poor life expectancy. Nonetheless, in the present study, we safely drained the pyothorax cavity near the central part of the thorax using a pigtail catheter under CT guidance, and significantly improved the patient's condition. The advantage of pigtail catheters is that they are less invasive than conventional thick trocar catheters. Pigtail catheters are thin and flexible, allowing them to reach deeper into the thoracic cavity. Minimally invasive drainage is desirable in very elderly patients, such as those over 90 years of age, patients with poor ADL, and patients with somebody contractures, as in the present case. On the other hand, since the target of treatment is pyothorax, catheters that are too thin (e.g., for venopuncture catheter) often become blocked internally with pus and cannot be drained properly. For this reason, a pigtail catheter was judged to be minimally invasive and just thin enough to allow drainage. Although larger drains are generally preferred for pyothorax,^4^ pigtail catheters may be a viable alternative, depending on the anatomical positioning and the patient's condition. Some argue that smaller drainage tubes have fewer complications and cause less pain and should be used for initial treatment.^5^

One of the disadvantages of pigtail catheters is their propensity for internal obstruction. Daily flushing of the route with heparin or saline and continuous negative pressure suction are therefore effective for preventing catheter blockage when used for pyothorax. Our goal in this case is not to highlight its rarity but rather its educational significance. Pyothorax is often a hesitant therapeutic intervention in the very elderly. Nevertheless, with the appropriate technique and ingenuity, it is sometimes possible to restore these patients early enough.

In conclusion, we successfully treated pyothorax in a very elderly patient using a pigtail catheter. We demonstrated that even very elderly patients with reduced cognitive function and ADL can be effectively treated with drainage, provided the right techniques are employed.

## AUTHOR CONTRIBUTIONS

Kohei Fujita, Masataka Hirai, and Zentaro Saito cared the patient. Takanori Ito, Takuma Imakita, Issei Oi, and Osamu Kanai advised the management of patient care. Hiromasa Tachibana and Tadashi Mio supervised the clinical management. Kohei Fujita drafted and all authors revised manuscript. All authors approved this manuscript for submission.

## CONFLICT OF INTEREST STATEMENT

None declared.

## ETHICS STATEMENT

The authors declare that appropriate written informed consent was obtained for the publication of this manuscript and accompanying images from the patient's eldest daughter.

## Data Availability

The data that support the findings of this study are available on request from the corresponding author. The data are not publicly available due to privacy or ethical restrictions.
